# Hypoxia and Macrophages Act in Concert Towards a Beneficial Outcome in Colon Cancer

**DOI:** 10.3390/cancers12040818

**Published:** 2020-03-28

**Authors:** Flávia Martins, Rosa Oliveira, Bruno Cavadas, Filipe Pinto, Ana Patrícia Cardoso, Flávia Castro, Bárbara Sousa, Marta Laranjeiro Pinto, Ana João Silva, Diogo Adão, José Pedro Loureiro, Nicole Pedro, Rui Manuel Reis, Luísa Pereira, Maria José Oliveira, Angela Margarida Costa

**Affiliations:** 1 i3S-Institute for Research and Innovation in Health, University of Porto, 4200-135 Porto, Portugal; 2 INEB-Institute of Biomedical Engineering, University of Porto, 4200-135 Porto, Portugal; 3 IPATIMUP-Institute of Molecular Pathology and Immunology, University of Porto, 4200-135 Porto, Portugal; 4 Department of Pathology and Oncology, Faculty of Medicine, University of Porto, 4200-319 Porto, Portugal; 5 ICBAS-Institute of Biomedical Sciences Abel Salazar, University of Porto, 4050-313 Porto, Portugal; 6 CNC-Center for Neuroscience and Cell Biology, University of Coimbra, 3004-517 Coimbra, Portugal; 7 IBMC-Institute of Molecular and Cellular Biology, University of Porto, 4200-135 Porto, Portugal; 8 Molecular Oncology Research Center, Barretos Cancer Hospital, 14.784-400 Barretos-SP, Brazil; 9 Life and Health Sciences Research Institute (ICVS), School of Medicine, University of Minho, 4710-057 Braga, Portugal; 10 ICVS/3B’s-PT Government Associate Laboratory, 4710-057 Braga, Portugal

**Keywords:** hypoxia, macrophages, colon cancer, tumor microenvironment, immune cell infiltration, prognosis

## Abstract

In colon cancer, the prognostic value of macrophages is controversial, and it is still unknown how hypoxia modulates macrophage–cancer cell crosstalk. To unravel this, co-cultures of human primary macrophages and colon cancer cells were performed at 20% and 1% O_2_, followed by characterization of both cellular components. Different colon cancer patient cohorts were analyzed for hypoxia and immune markers, and their association with patient overall survival was established. A positive correlation between *HIF1A* and *CD68* in colon cancer patients was identified but, unexpectedly, in cases with higher macrophage infiltration, *HIF1A* expression was associated with a better prognosis, in contrast to breast, gastric, and lung cancers. Under hypoxia, co-cultures’ secretome indicated a shift towards a pro-inflammatory phenotype. These alterations occurred along with increased macrophage phagocytic activity and decreased SIRPα expression. Cancer cells were more invasive and exhibited higher CD47 expression. We hypothesize that the better prognosis associated with *HIF1A*^High^*CD68*^High^ tumors could occur due to macrophagic pro-inflammatory pressure. Indeed, we found that tumors *HIF1A*^High^*CD68*^High^ expressed increased levels of *CD8A*, which is positively correlated with *HIF1A*. In conclusion, we show that in colon cancer, hypoxia drives macrophages into a pro-inflammatory phenotype, concomitant with increased infiltration of anti-tumor immune cells, favoring better disease outcome.

## 1. Introduction

It is recognized that the non-malignant component of the tumor microenvironment (TM) has a pivotal role in both tumorigenesis and tumor progression. To improve cancer therapy, it is crucial to better understand cancer cell behavior and the associated molecular mechanisms, which cannot be achieved without knowing how the different TM components influence each other’s behavior, driving disease outcomes. As an ecosystem, the TM is composed of different entities besides cancer cells, namely, immune cells, fibroblasts, adipocytes, and the extracellular matrix (ECM), which communicate and modulate each other’s activities [1].

Macrophages are abundant in the TM, and their high infiltration is associated, in several malignancies, with poor prognosis and therapy resistance [2]. Due to their plasticity, macrophages can polarize into pro-inflammatory and anti-tumor or into anti-inflammatory and pro-tumor [3], being, therefore, interesting therapeutic targets. In fact, it is known that a pro-inflammatory environment is a driving force for the tumorigenic process, driving mutagenesis, while in the context of an established tumor, the pro-inflammatory environment could favor the elimination of already-transformed cells [4].

In colon cancer, the role of macrophages is controversial, with some authors claiming that a high infiltration is associated with better prognosis, while others report that their presence is associated with increased tumor progression-associated activities [5]. Our group has previously evidenced that macrophages induce gastric and colon cancer cell invasion, in a process partially mediated by secreted factors, and that the secretome of macrophages differs from that of macrophages co-cultured with cancer cells [6].

Due to aberrant cancer cell growth and abnormal vascularization, the TM is generally hypoxic. This condition leads to reactive oxygen species accumulation and acidosis, both associated with enhanced invasion, metastasis, and therapy resistance [7], hypoxia being an independent prognostic factor in several types of cancer [8]. Nevertheless, the mechanisms through which hypoxia interferes with the non-malignant components of TM are still a matter of study.

It has been reported that macrophages preferentially accumulate within tumor hypoxic regions, and that hypoxia leads to the genetic reprogramming of both cancer cells and macrophages, mainly through hypoxia inducible factor (HIF) transcription factors. HIF1α, the most important of them, is constitutively expressed and is targeted to degradation at normal oxygen tension, but stabilized under hypoxia [9,10]. The activation of HIF downstream targets leads to cellular adjustments such as metabolic alterations and expression of ECM-remodeling enzymes [11]. It is also known that HIF can affect the immunogenicity of hypoxic breast cancer by inducing the expression of CD47, a cell surface receptor that impairs macrophage pro-phagocytic signaling [12]. Due to its importance, HIF-inhibitors are under consideration as anti-cancer agents [13]. However, the experiments studying the colon cancer cells–macrophages interplay were not performed under hypoxia, nor were the studies about hypoxia focused on this crosstalk, resulting in an incomplete understanding of the role of hypoxia in the colon cancer microenvironment. Giving the controversial role of macrophages in colon cancer, the study of this interplay will provide a deeper understanding of the colon cancer microenvironment complexity.

In the present study, we characterize the effect of low oxygen levels on macrophage behavior and the modulation of macrophages and cancer cells crosstalk. The influence of hypoxia and macrophages is also analyzed in different cohorts of colon cancer patients. The data gathered in this work increases the insight on colon TM specificity and provides information that can result in the development of novel and more efficient therapeutic strategies.

## 2. Results

### 2.1. CD68 and HIF1A Are Positively Correlated in Colon Cancer Patients, and the Population CD68^High^HIF1A^High^ Presents Better Prognosis

**Figure 1:**
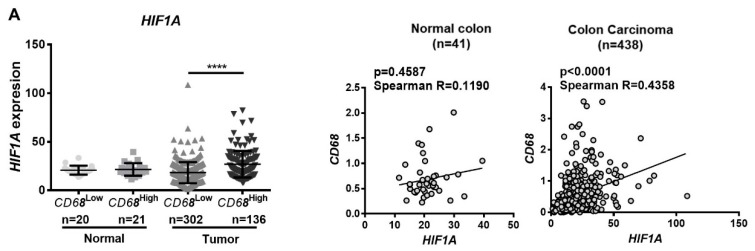

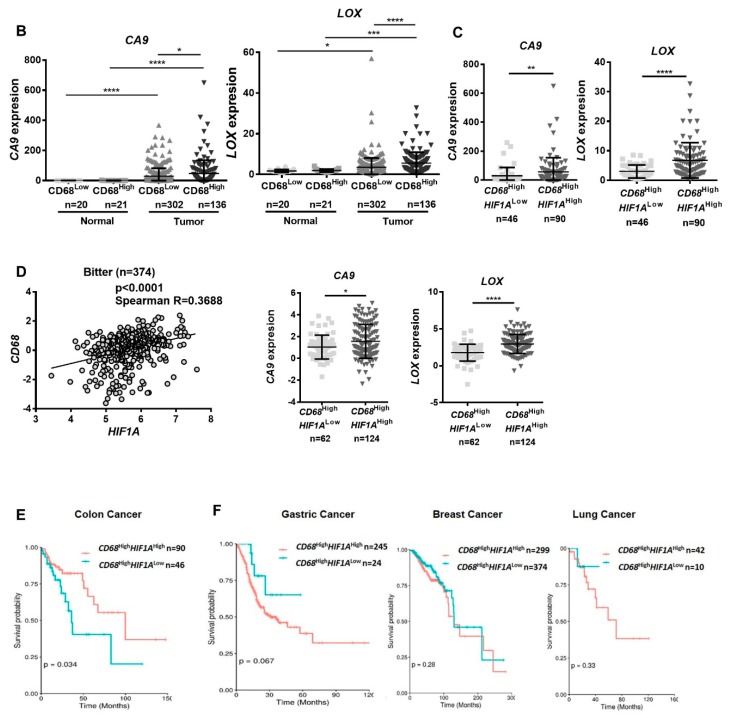
*CD68* and *HIF1A* are positively correlated in colon cancer, and *CD68^High^HIF1A^High^* tumors present better prognosis. Microarray and RNASeq expression data were downloaded from The Cancer Genome Atlas (TCGA) (**A**–**C**, **E**–**F**) and from Oncomine databases (**D**). (**A**–**C**) Normal and tumor samples were divided into *CD68*^Low^ and *CD68*^High^, according to the median levels of *CD68* expression in normal samples. In each group, the expression of the hypoxic marker *HIF1A* (**A**), *CA9* and *LOX* (**B**,**C**) genes, known to be regulated by HIF1α in hypoxic conditions, was evaluated. (**A**) The correlation between *CD68* and *HIF1A* was assessed. (**B**,**C**) The expression of *CA9* and *LOX* was evaluated in both normal and tumor samples, in *CD68*^High^ and *CD68*^Low^ populations, and within the *CD68*^High^ population, the expression levels of both genes were compared between *HIF1A*^Low^ and *HIF1A*^High^, according to the median levels of *HIF1A* expression in normal samples. (**D**) Correlations between *CD68* and *HIF1A*, and *LOX* and *CA9* expression in *CD68*^High^*HIF1A*^High^ patients were confirmed on Bittner’s colon cancer cohort from Oncomine. (**E**,**F**) Kaplan–Meier curves were made with patients’ survival data, comparing the populations *CD68*^High^*HIF1A*^High^ and *CD68*^High^*HIF1A*^Low^. The division high/low expression was made according to the median expression of the selected genes in normal tissue (**A**–**C**; **E**–**F**), or by the median expression of tumor (**D**). Data from colon (**E**), gastric, breast, and lung (**F**) cancer patients were analyzed. Kruskal–Wallis, Mann–Whitney, or unpaired *t*-test were used to compare gene expression among groups. Spearman’s statistical test was used to assess correlation, which was considered moderate positive when Spearman Rs were between 0.2500 and 0.3500, and strong positive when Spearman R > 0.3500. Long-rank (Mantel–Cox) test was performed in the analysis of the survival curves. **** *p* < 0.0001; *** *p* < 0.001; ** *p* < 0.01; * *p* < 0.05.

To understand the relevance of hypoxia on the modulation of macrophage behavior in colon cancer patients, we took advantage of TCGA and Oncomine databases. Therefore, the expression levels of *CD68*, a macrophage lineage marker, and of *HIF1A*, a master hypoxia regulator, in which slight mRNA changes can cause significant alterations in its activity and protein levels, were analyzed [14]. Since TCGA compiles expression data from normal and cancer colon tissues, we focused our analysis in *HIF1A* in *CD6*8^High^ and *CD68^Low^* populations, in both tissues, assuming that *CD68^High^* population has a higher degree of macrophagic infiltration. As normal colon tissue could have some endogenous degree of inflammation, the threshold used to subdivide the population between *CD68*^High^ and *CD68*^Low^ was defined based on the median *CD68* expression in the normal tissue. Moreover, we found that *HIF1A* expression was significantly higher in tumors from patients with elevated macrophage infiltration (*CD68*^High^) and that *CD68* expression positively correlated with *HIF1A* expression (Figure 1A). It is described that colon carcinomas exhibit increased nuclear expression of HIF1-α [15]. However, as it may be rapidly targeted to degradation, we evaluated the expression levels of HIF1-α downstream targets that are upregulated under hypoxia conditions, specifically *CA9* and *LOX* (Figure 1B). Using the previous approach, we found a significant increase in *CA9* and *LOX* expression in tumor tissue, which was significantly more pronounced in *CD68*^High^ patients (Figure 1B). Due to the present focus on the role of macrophages on the TM, we particularly evaluated the *CD68*^High^ population. By dividing this population between *HIF1A*^High^ and *HIF1A*^Low^, we found that *CA9* and *LOX* expression levels are significantly higher in the *HIF1A*^High^ population (Figure 1C). The positive correlation between *CD68* and *HIF1A*, and the increased *CA9* and *LOX* expression in *CD68^High^HIF1A^High^* patients were validated on the Oncomine database (colon cancer Bittner cohort, with the highest number of patients) (Figure 1D). *CA9* or LOX were not used in further analysis as hypoxic markers due to statistical constraints when the number of patients needed to be sub-divided into *CD68*^High^ and *CD68*^Low^, and *HIF1A*^High^ and *HIF1A*^Low^.

A major point of interest was to investigate whether the association between high macrophage infiltration and enhanced expression of hypoxic markers had any impact on patient prognosis. Surprisingly, we found that the *CD68*^High^*HIF1A*^High^ population presented a significantly better prognosis than the CD68^High^*HIF1A*^Low^ population (Figure 1E). To check whether this was a common feature in cancers where macrophagic infiltration was associated with prognosis, we used the same TCGA approach to analyze gastric, breast, and lung cancer patients. Despite the low number of patients in some populations, this specific association only occur in colon cancer (Figure 1F). The same analysis was not performed in the Oncomine cohort since survival data is not available.

### 2.2. Hypoxia Impacts Macrophage Antigen-Presentation Associated Molecules

In order to understand the impact of the hypoxic microenvironment on macrophage–colon cancer cell crosstalk, and particularly on macrophage function, indirect co-cultures of macrophages with cancer cells were established at both 20% and 1% O_2_ (Figure S1A). The significant increase in cancer cell *CA9* expression at 1% O_2_ validates the cellular sensing of the hypoxic stimulus, to which both cells were simultaneously exposed to (Figure S1B), without affecting cellular viability between the conditions (Figure S1C). This indirect co-culture system enables the analysis of differences induced by exchanged secreted factors, but in conditions that allow the full recovery of both cellular populations for further independent studies. The selection of primary macrophages instead of the widely used and modified human monocytic cell line, THP-1, added variability to our equation, being more representative of the existent individual differences. Besides more closely mimicking the TM, human macrophages were preferred in relation to their murine counterparts, given the described interspecies variability regarding polarization markers and activation programs [16].

**Figure 2:**
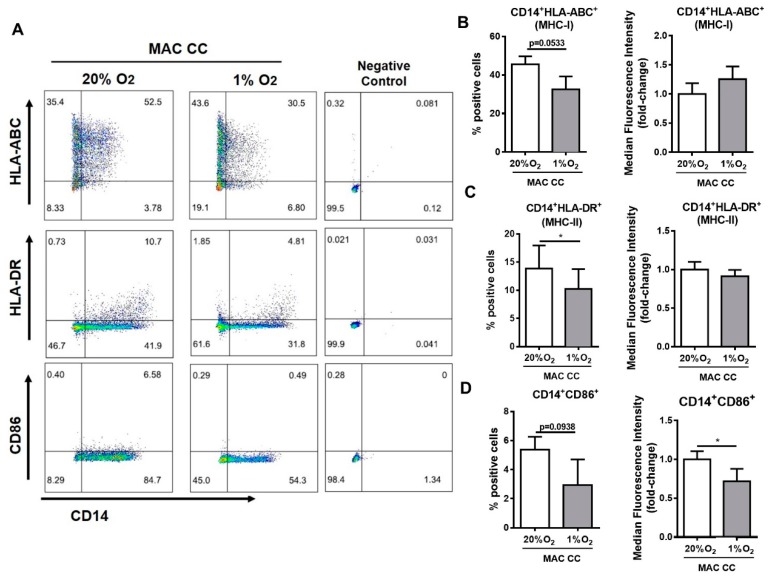
Hypoxia impacts macrophage antigen-presentation associated molecules. Macrophages were indirectly co-cultured with colon cancer cells RKO (MAC CC), at 20% or 1% O_2_ for 72 h. Expression of the monocyte/macrophage lineage marker CD14, HLA-ABC (MHC-I), HLA-DR (MHC-II), and CD86 was determined by flow cytometry. (**A**) Pseudo-color plots display the gating strategy for flow cytometry created with FlowJo. (**B**–**D**) Graphs represent the percentage and median fluorescence intensity, which is presented as fold-change relatively to 20% O_2_ condition of (**B**) CD14+HLA-ABC+, (**C**) CD14+HLA-DR+, or (**D**) CD14+CD86+ cells. Graphs represent the mean values with standard deviations, and are representative of *n* = 7 (HLA-ABC); *n* = 10 (HLA-DR); *n* = 6 (CD86) independent experiments. The statistical tests Wilcox or paired *t*-test were used; * *p* < 0.05. The *p*-values between 0.05 and 0.1 were presented and considered a tendency.

Macrophages are professional antigen-presenting cells, and immune evasion of cancer cells is a major cancer hallmark. To analyze whether hypoxia can trigger some alterations in the expression of key molecules involved in antigen presentation, macrophage major histocompatibility complexes (MHC) class I (HLA–ABC) and class II (HLA–DR) molecules, and of the co-stimulatory receptor CD86, were investigated by flow cytometry (gating strategy described in Figure S2A). Our results at 1% O_2_ evidenced a slight reduction in the percentage of macrophages expressing HLA–ABC (*p* < 0.0533) and CD86 (*p* < 0.0938) (Figure 2A,B,D), with a significant decrease in the percentage of cells expressing HLA–DR (Figure 2A,C) and in the intensity of CD86 expressed by each cell (Figure 2A,D).

### 2.3. Hypoxia Modulates Macrophage Phagocytic Activity and SIRPα Expression, and CD47 Expression on Cancer Cells

One of the major functions of macrophages is to phagocyte foreign or self-cellular components targeted to degradation. This is particularly important in the context of macrophage defensive functions against pathogens, their role in regeneration, and also in anti-tumor activities. Noteworthy, it is known that cancer cells express “don’t eat me” signals, as the CD47 receptor, which will bind to SIRPα in macrophages, and impairs phagocytosis.

**Figure 3:**
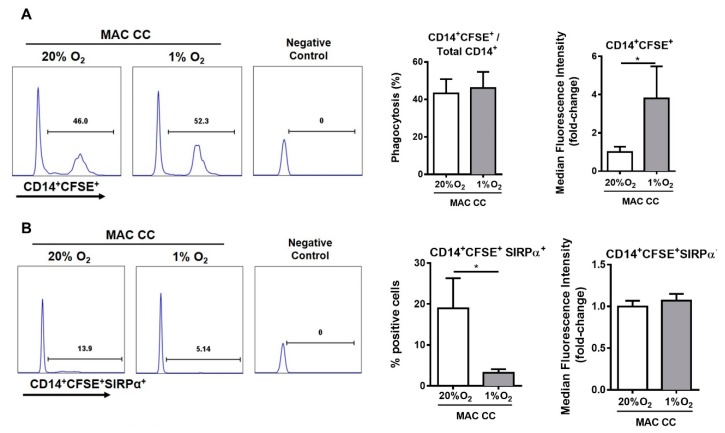

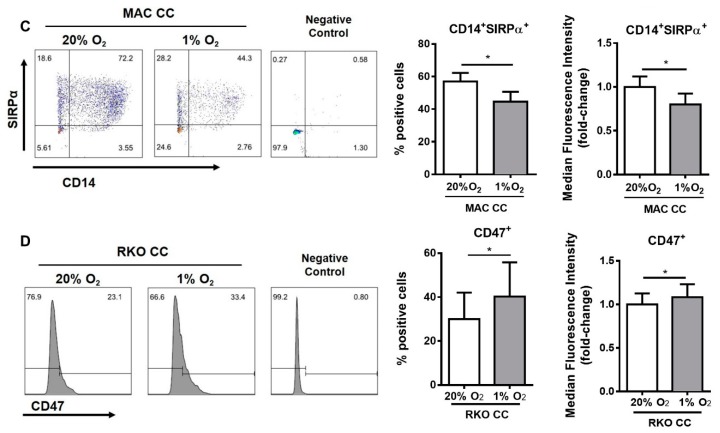
Hypoxia modulates macrophage phagocytic activity and SIRPα expression and CD47 expression on cancer cells. (**A**,**B**) Macrophages that were indirectly co-cultured with RKO (MAC CC) cells, at 20% or 1% O_2_ for 72 h, were posteriorly directly stimulated with CFSE-labeled RKO cells for 2 h. Macrophages were analyzed by flow cytometry. Histograms display the gating strategy for flow cytometry created with FlowJo. (**A**) Phagocytic activity was calculated as the ratio of CD14+CFSE+ cells/Total CD14+ cells. (**B**) Graphs represent the percentage of CD14+CFSE+SIRPα+ cells, and the median fluorescence intensity, which is presented as fold-change relatively to 20% O_2_ condition. (**C**,**D**) Macrophages were indirectly co-cultured with RKO, at 20% or 1% O_2_ for 72 h. Expression of the monocyte/macrophage lineage marker CD14 and SIRPα was evaluated on macrophages (**C**) and of CD47 on cancer cells (**D**) by flow cytometry. Pseudo-color plots (**C**) and histograms (**D**) display the gating strategy for flow cytometry. Graphs represent the percentage and median fluorescence intensity, which is presented as fold-change relatively to 20% O_2_ condition of (**C**) CD14+SIRPα+, or (**D**) CD47 cells. Graphs represent the mean values with standard deviations, and are representative of *n* = 11 (phagocytosis); *n* = 7 (CFSE+SIRPα+); *n* = 10 (SIRPα); *n* = 11 (CD47) independent experiments. The statistical tests Wilcox or paired *t*-test were used; * *p* < 0.05.

In the present study, we exploited whether hypoxia alters macrophage phagocytic activity, in a similar process already described in breast cancer [12], using hypoxic cancer cells as a phagocytic stimulus. Accordingly, macrophages were co-cultured with cancer cells for 72 h at 20% or 1% O_2_ (Figure S1A). Then, cancer cells were removed, and macrophages were directly stimulated with CFSE (Carboxyfluorescein Diacetate Succinimidyl Ester)-labeled cancer cells, previously cultured at 1% O_2_ for 72 h. Although no differences were observed in the percentage of phagocytic macrophages, the amount of phagocyted CFSE-labeled cancer cells almost doubled on macrophages pre-conditioned by hypoxia and cancer cells (Figure 3A and Figure S2A,B). Afterwards, we explored alterations in SIRPα levels in the phagocytic macrophages, those engulfing CFSE+ cancer cells (CD14+CFSE+). Interestingly, we found a significant decrease in the percentage of phagocytic macrophages expressing SIRPα, when previously maintained at 1% O_2_ in comparison to those at 20% O_2_ (Figure 3B and Figure S2A,B). Based on these results, we further analyzed the SIRPα expression in macrophages and CD47 in cancer cells from the indirect co-cultures, maintained for 72 h at 20% or 1% O_2_, before the direct contact with the CFSE-labeled cancer cells. Notably, we found that the co-cultures were already sufficient to trigger a decrease in macrophage SIRPα (Figure 3C), and an increase in cancer cells CD47 (Figure 3D).

### 2.4. Hypoxia Decreases the Anti-Inflammatory Pressure and Increases IL-1β Secretion

**Figure 4:**
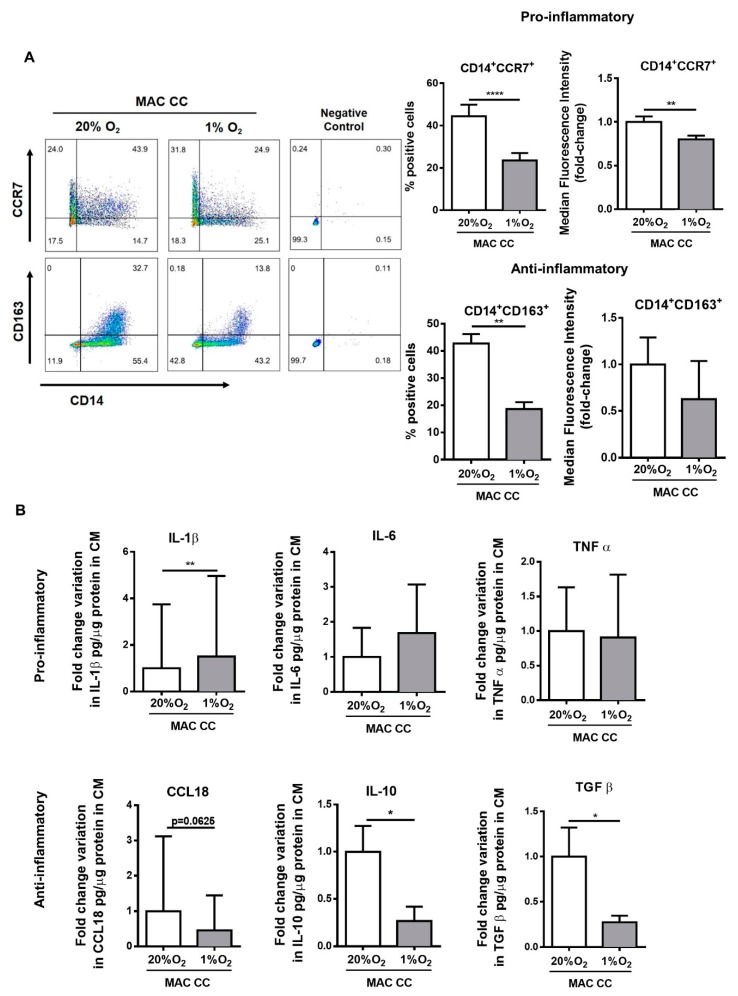
Hypoxia decreases the anti-inflammatory pressure and increases IL-1β secretion. Macrophages were indirectly co-cultured with RKO (MAC CC), at 20% or 1% O_2_ for 72 h. (**A**) Expression of CD14 and of the polarization markers CCR7 and CD163 was evaluated on macrophages by flow cytometry. Pseudo-color plots display the gating strategy for flow cytometry. Graphs represent the percentage and median fluorescence intensity, which is presented as fold-change relatively to 20% O_2_ condition. (**B**) Concentration of secreted molecules was measured by ELISA, and normalized to conditioned media (CM) protein levels. Graphs represent cytokine concentration of pro- and anti-inflammatory markers, as a fold change variation to macrophages cultured at 20% O_2_. Graphs display the mean values with standard deviations, and are representative of *n* = 12 (CCR7); *n* = 5 (CD163); *n* = 10 (IL-1β); *n* = 8 (IL-6); *n* = 5 (TNF-α); *n* = 5 (CCL18); *n* = 5 (IL-10); *n* = 7 (TGF-β) independent experiments. The statistical tests Wilcox or paired *t*-test were used **** *p* < 0.0001; ** *p* < 0.01; * *p* < 0.05. The *p*-values between 0.05 and 0.1 were presented and considered a tendency.

It is described that, depending on the stimulation, macrophages may polarize as pro- or anti-inflammatory, sustaining thereafter anti- or pro-tumor activities, respectively [3]. Therefore, we investigated the impact of hypoxia on the polarization profile of macrophages co-cultured with cancer cells, analyzing the expression of a panel of pro- and anti-inflammatory markers [17] by flow cytometry and ELISA. Despite the fact that hypoxia is usually associated with a worse prognosis and with cellular features related to tumor progression, we found that 1% O_2_ treatment resulted in a mixed macrophage phenotype, with alterations in both pro and anti-inflammatory markers. However, the clear decrease in anti-inflammatory markers CD163, TGFβ, CCL18, and IL-10 (Figure 4A,B), along with the increased expression of the pro-inflammatory cytokine IL-1β (Figure 4B), which seems to sustain a hypoxia-induced pro-inflammatory effect, is relevant. Nevertheless, we also observed a significant decrease in pro-inflammatory marker CCR7 (Figure 4A).

### 2.5. Hypoxia Impacts Colon Cancer Cell Behavior, Increasing Invasion and Glucose Consumption

Given the alterations in macrophage behavior induced by the hypoxic environment, as well as differences regarding a patient’s overall survival, we decided to assess whether hypoxia and macrophages influence cancer cell behavior.

**Figure 5:**
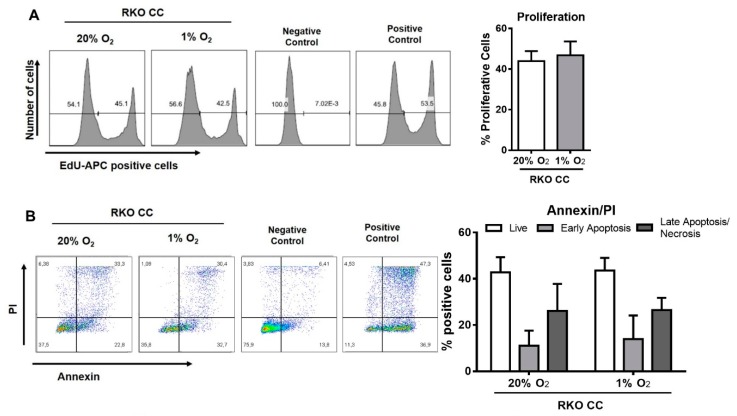

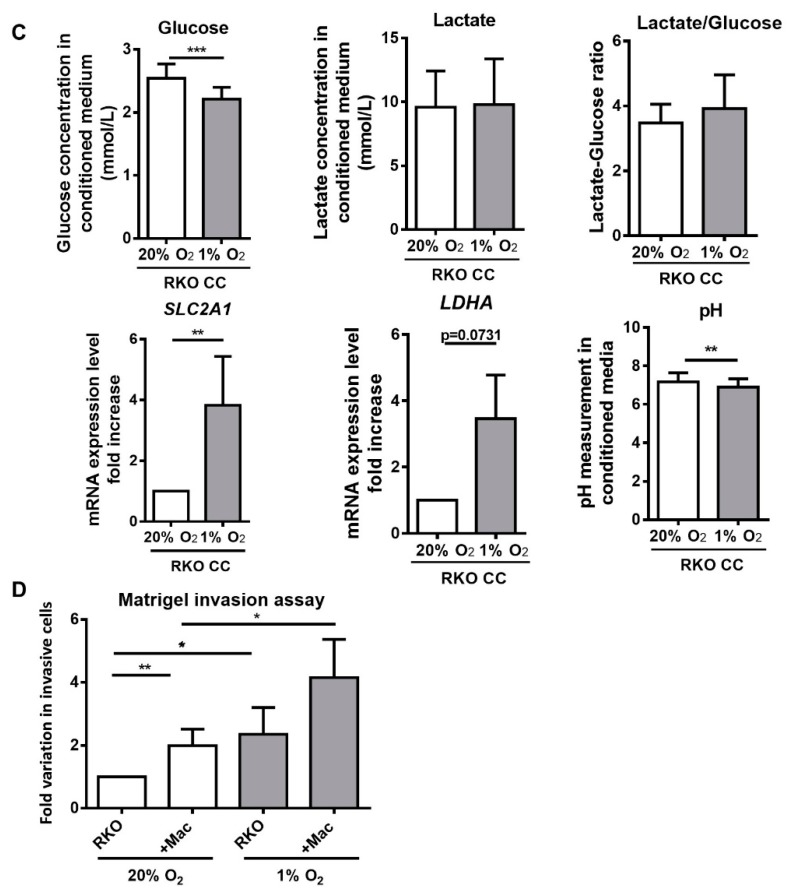
Hypoxia impacts colon cancer cell behavior, increasing invasion and glucose consumption. (**A**–**C**) Macrophages were indirectly co-cultured with RKO, at 20% or 1% O_2_ for 72 h, and (**A**) proliferation of cancer cells was assessed for that period. Data are presented as percentage of proliferative cells. (**B**) AnnexinV/PI assay was used to quantify the percentage of viable (AnnexinV/PI negative), early apoptotic (AnnexinV positive/PI negative), and late apoptotic/necrotic (Annexin V/PI positive) cells. (**C**) Lactate production, glucose consumption, and pH measurements were made on co-cultures CM. Lactate/glucose ratio was calculated with the glucose and lactate concentration values of the same experiments. Cancer cells *SLC2A1* and *LDHA* mRNA levels were measured by qRT–PCR. Relative expression changes are presented as fold variation *SLC2A1*/*ACTB* and *LDHA/ACTB* relatively to 20% O_2_ condition. (**D**) Cells maintained at 20% or 1% O_2_, and stimulated or not with macrophages previously maintained at 20% or 1% O_2_ were cultured for 24 h on Matrigel coated filters, at 20% or 1% O_2_. Data are presented as fold variation in the number of invasive cells relative to RKO cells cultured at 20% O_2_ without any stimulation with macrophages. Graphs represent the mean values with standard deviations, and are representative of *n* = 5 (proliferation assay); *n* = 6 (Annexin V/PI assay); *n* = 10 (glucose quantification); *n* = 11 (lactate quantification); *n* = 6 (*SLC2A1*); *n* = 5 (*LDHA*); *n* = 9 (pH); *n* = 10 (invasion assay) independent experiments. The statistical tests Friedman, RM one-way ANOVA, Wilcox or paired *t*-test were used *** *p* < 0.001; ** *p* < 0.01; * *p* < 0.05. The *p*-values between 0.05 and 0.1 were presented and considered a tendency.

No differences were observed in both cell proliferation and cell death in cancer cells when co-cultured with macrophages at 20% or 1% O_2_ (Figure 5A,B).

As it is well reported that hypoxia can modulate cancer cell metabolism favoring glycolysis, we evaluated the levels of glucose consumption, lactate production, and pH in indirect co-cultures media, established at 20% and 1% O_2_. It is important to note that the glucose and lactate levels in co-cultures conditioned media (CM) results from both macrophages and cancer cells, being impossible to discriminate the specific contribution of each cellular population. To obtain additional information, cancer cell expression levels of *SLC2A1*, the gene that codifies the glucose transporter GLUT-1, and of *LDHA*, the gene that codifies an enzyme involved in lactate production were also analyzed. Interestingly, at 1% O_2_, we found a significant increase in glucose consumption, which was accompanied by enhanced expression of the glucose-transporter codifying gene (*SLC2A1*) (Figure 5C). Nevertheless, no differences in lactate production were observed, despite the increase in *LDHA* expression, as well as a significant decrease in pH levels (Figure 5C). Noteworthy, all the differences found resulted from the combination of both macrophages and hypoxia.

When we looked into the invasive capacity of cancer cells, we observed that macrophages and hypoxia alone were sufficient to promote cancer cell invasion and, most importantly, this is potentiated when both factors are combined (Figure 5D).

### 2.6. Colon Cancer Patients with CD68^High^HIF1A^High^ Phenotype Present Increased Expression of Pro-Inflammatory Cytokines and Iincreased Infiltration of Cytotoxic T-Cells

While the in vitro results indicated that hypoxia was associated with features related both with worse prognosis (increased invasion, decreased expression of macrophage antigen-presenting molecules) and good prognosis (increase in phagocytosis and secretion of pro-inflammatory cytokines), the colon cancer patient’s survival data evidenced that the *CD68*^High^*HIF1A*^High^ population correlates with better prognosis. These led us to hypothesize that the in vitro cellular features associated with better prognosis may prevail in vivo, particularly the secretion of pro-inflammatory cytokines, which could lead to the recruitment of other anti-tumor immune cells. 

**Figure 6:**
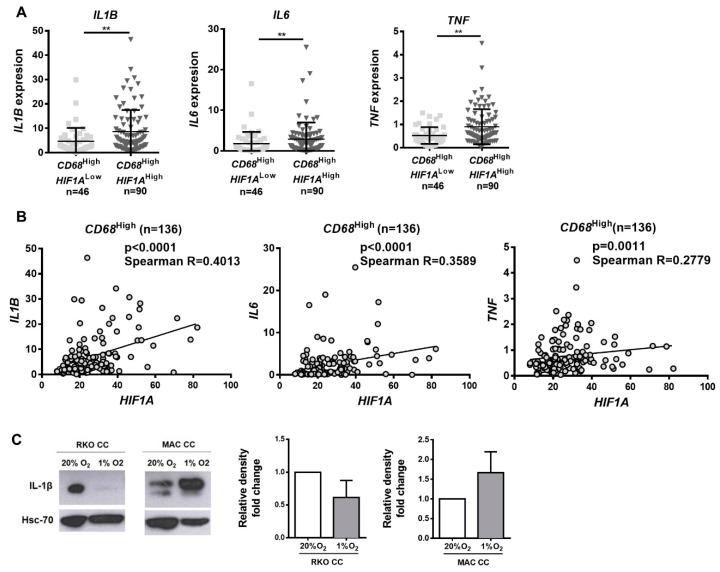

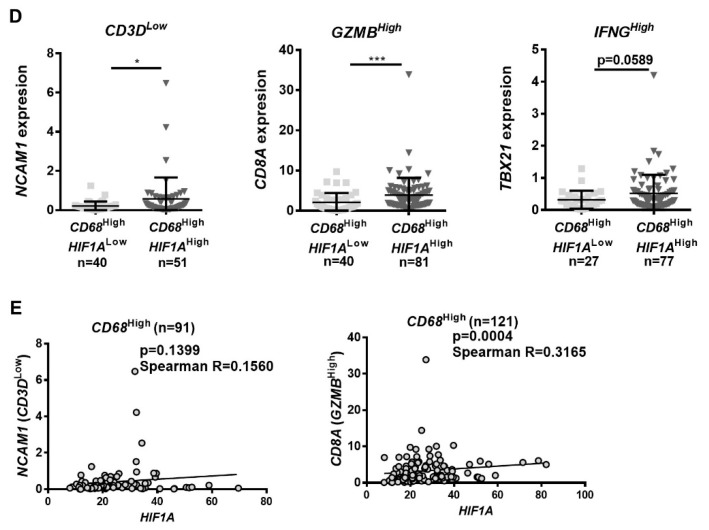
*CD68^High^HIF1A^High^* tumors present increased expression of pro-inflammatory cytokines and increased infiltration of cytotoxic T-cells. RNASeq expression data were downloaded from TCGA. (**A**) The expression of *IL1B*, *IL6*, and *TNF* was evaluated within the *CD68*^High^ population, between *HIF1A*^Low^ and *HIF1A*^High^, according to the median levels of *CD68* and *HIF1A* expression in normal samples. (**B**) The correlation between *IL1B*, *IL6*, *TNF*, and *HIF1A* was assessed within the *CD68*^High^ population. (**C**) IL-1β protein levels were evaluated in total lysates from co-cultured RKO cells (RKO CC) and macrophages (MAC CC), by Western blot, and band densitometry quantification presented. Hsc70 was used as loading control, and the figure is representative of 3 independent experiments. (**D**) The expressions of *NCAM1*, *CD8A*, and *TBX21* were evaluated within the *CD68*^High^ population, between *HIF1A*^Low^ and *HIF1A*^High^ subpopulations, according to the median levels of *CD68* and *HIF1A* expression in normal samples. *NCAM1* levels were evaluated within the *CD3D*^Low^ population, *CD8A* levels were evaluated within the *GZMB*^High^ population, and *TBX21* levels were evaluated within the *IFNG*^High^ population. *CD3D*^Low^, *GZMB*^High^, and *IFNG*^High^ populations were established according to the median levels of each marker expression in normal samples. (**E**) Correlations between *NCAM1*(*CD3D*^Low^), *CD8A*(*GZMB*^High^), and *HIF1A* were assessed within the *CD68*^High^ population. Mann–Whitney statistical test was used to compare expression between groups. Spearman statistical test was used to assess correlation, which was considered moderate positive when Spearman Rs were between 0.2500 and 0.3500, and strong positive when Spearman R > 0.3500. **** *p* < 0.0001; *** *p* < 0.001; ** *p* < 0.01; * *p* < 0.05. The *p*-values between 0.05 and 0.1 were presented and considered a tendency.

Initially, the expression of the three pro-inflammatory cytokines previously analyzed, IL-1β, IL-6, and TNF-α, were compared between *CD68^High^HIF1A^High^* and *CD68*^High^*HIF1A*^Low^ patients, using the TCGA and Oncomine databases. A significantly increased expression of the three cytokines was confirmed in *CD68*^High^*HIF1A*^High^ tumors (Figure 6A). Moreover, in *CD68*^High^ tumors, a positive correlation between *HIF1A* and the expression of the three pro-inflammatory cytokines was found (Figure 6B). These results corroborate our in vitro findings, in which the analysis of the total lysates of macrophages or cancer cells exposed to 20% or 1% O_2_ for 72 h evidenced that the main contributor to the pro-inflammatory IL-1β cytokine, detected in co-cultures CM, is indeed the macrophages and not the cancer cells (Figure 6C and Figure S4). Since it is known that a pro-inflammatory pressure can contribute to the recruitment and activation of anti-tumor immune cells [18,19,20], we assessed whether *CD68*^High^*HIF1A*^High^ enriched tumors presented an increased expression of markers of natural killer (NK) cells *(NCAM1*, within the *CD3D*^Low^ population), cytotoxic T-cells (*CD8A*, within the *GZMB*^High^ population), and Th1 cells (*TBX21*, within the *IFNG*^High^ population). Our results confirmed a significant increase in the expression of *NCAM1* and *CD8A*, and an increase, although not statistically significant, of *TBX21* (Figure 6D). Nevertheless, of the two markers that presented significant differences, only CD8A evidenced a positive correlation with *HIF1A* expression on the *CD68*^High^ tumors (Figure 6E).

The results from the TCGA data analysis were confirmed using the Oncomine Bittner colon cancer cohort (Figure S3). Of note, since this cohort does not include data regarding *IFNG* expression, only the levels of *TBX21*, a Th1 cell-specific transcription factor which controls the expression of the hallmark Th1 cytokine IFN-γ was used.

### 2.7. The Pressure of Hypoxia on Macrophages at the CMS1-MSI-Immune Colon Cancer Type

**Figure 7:**
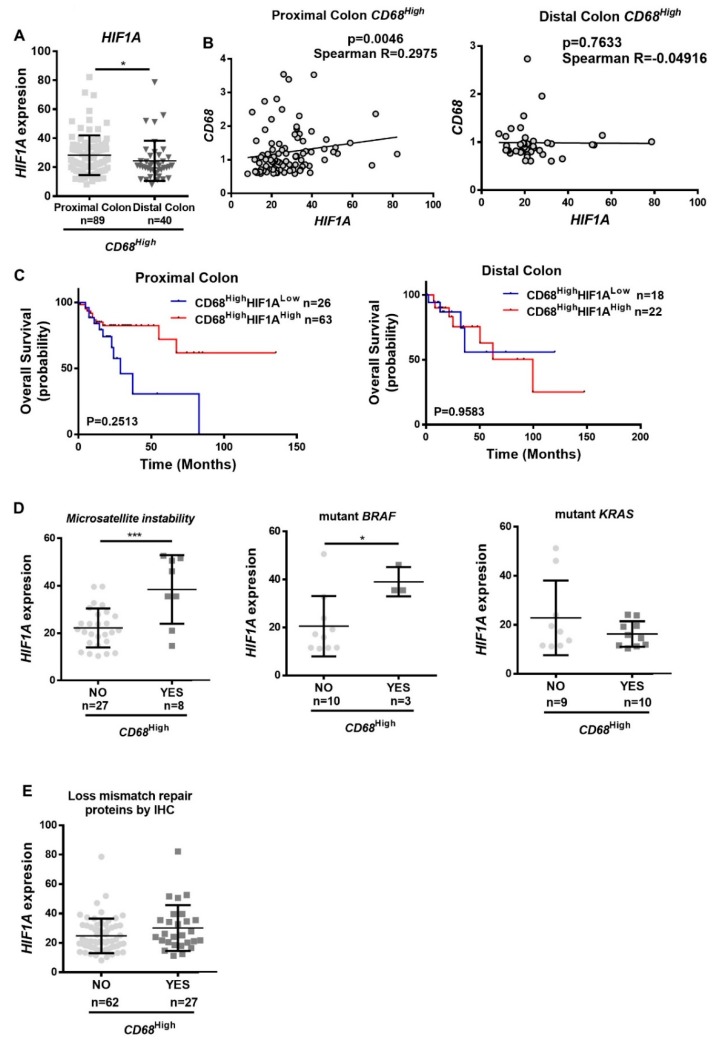
The pressure of hypoxia on macrophages at the consensus molecular subtype 1–microsatellite instable (CMS1–MSI)-immune colon cancer type. Microarray and RNASeq expression data were downloaded from TCGA. (**A**) *HIF1A* expression was evaluated between patients with tumors in proximal colon (cecum, ascending colon, hepatic flexure, and transverse colon) and in distal colon (splenic flexure, descending colon, and sigmoid colon), within the *CD68*^High^ population. (**B**) The correlation between *CD68* and *HIF1A* was assessed in proximal and distal colon population. (**C**) Kaplan–Meier curves were made with patients’ survival data, comparing the populations *CD68*^High^*HIF1A*^High^ with the population *CD68*^High^*HIF1A*^Low^, on distal and proximal colon patients. (**D**) *HIF1A* expression was evaluated, within the *CD68*^High^ population, between patients with or without microsatellite instability, BRAF and KRAS mutations. (**E**) *HIF1A* expression was evaluated, within the *CD68*^High^ population, between patients with or without loss of mismatch repair proteins, evaluated by immunohistochemistry. The division high/low expression was made according the median expression of the genes in normal tissue. Mann–Whitney statistical test was used to compare expression between groups. Spearman statistical test was used to assess correlation, which was considered moderate positive when Spearman Rs were between 0.2500 and 0.3500, and strong positive when Spearman R > 0.3500. Long-rank (Mantel–Cox) test to the analysis of the survival curves. ** *p* < 0.01; * *p* < 0.05.

We anticipated that the observed phenotype might be more relevant in the consensus molecular subtype 1–microsatellite instable (CMS1–MSI)-immune colon cancer subtype [21], due to the increased immune infiltration. To test our hypothesis, we compared the *HIF1A* expression in distal and proximal colon, within the tumors expressing *CD68*^High^, as the proximal colon is associated with the aforementioned colon cancer subtype. Accordingly, we found that *HIF1A* expression is significantly higher in the proximal than on the distal colon (Figure 7A). In addition, the correlation between *CD68* and *HIF1A* was only observed in proximal tumors (Figure 7B), supporting our hypothesis. Despite no significant differences being found on the survival rates of patients harboring *CD68*^High^*HIF1A*^High^ and *CD68*^High^*HIF1A*^Low^ proximal and distal colon tumors, it is possible to observe that the pattern between the two locations is clearly different, suggesting that *CD68*^High^*HIF1A*^High^ patients might have a better prognosis than *CD68*^High^*HIF1A*^Low^ patients (Figure 7C). This question should be clarified by increasing the sample size. Since the CMS1–MSI-immune colon cancer subtype is also characterized by high MSI, high levels of *BRAF* mutations, and low levels *KRAS* mutations, we decided to explore whether *CD68*^High^ tumors presented differences in *HIF1A* expression comparing MSI/MSS, and *BRAF* and *KRAS* wild-type versus mutated. Despite the low number of cases available at TCGA with the characterization of the MSI status and *BRAF* and *KRAS* mutations, our analysis showed that, in *CD68*^High^ tumors, the *HIF1A* expression is significantly higher in MSI and *BRAF* mutated cases (Figure 7D). Given these differences, we also looked to *HIF1A* expression in tumors with and without loss of mismatch repair proteins, but no significant difference was found (Figure 7E).

## 3. Discussion

The full understanding of the interactions established among the distinct TM components is crucial for the development of novel and more efficient anti-tumor therapies. The case of colon cancer is particularly relevant due to the high incidence and mortality, and because several studies have described a very particular behavior of colon cancer cells, in comparison with other tissues, underlying the importance of the colon cancer microenvironment.

In this work, we focused our attention on the crosstalk established between macrophages and colon cancer cells, and on its modulation by hypoxia. Hypoxia is a key feature of solid tumors that has been reported to critically influence cancer cell behavior and is often disregarded. In fact, most of the studies focusing on the role of hypoxia at the tumor microenvironment explored cancer cell metabolic changes [8], while its influence in the non-tumor compartment is still poorly explored. Therefore, the present work brings pioneer information concerning the study of hypoxia on the modulation of macrophage and colon cancer cell interactions.

When exploring tumor databases, we demonstrated that colon tumors exhibit distinct levels of macrophage infiltration, where the most infiltrated tumors are also the most hypoxic ones, presenting higher levels of *HIF1A*, *CA9,* and *LOX* expression. Although the novelty of these findings in colon cancer, these results are not unexpected since it has been previously reported, in other tumors, that hypoxic areas are the ones with enhanced inflammatory infiltration [9]. Notably, this association was accompanied by improved survival rates, clearly evident in *CD68*^High^ tumors, which could reinforce the idea of a protective anti-tumor and pro-inflammatory effect of the recruited macrophages. Indeed, it was previously reported that chemotactic HIF is essential for myeloid cell infiltration and activation through a VEGF-independent mechanism [22]. Here, we assume that *HIF1A* is produced by both tumor and non-tumor cells at the microenvironment, contributing to this immune cell recruitment, but it will be important to determine which population is the main contributor to pursue possible therapeutic applications. It will also be interesting to evaluate the role of HIF2α in this process, as it was reported that HIF1α and HIF2α have opposing roles [23]. In contrast to our results, previous reports in colon cancer described that HIF1α had no association [24], or was associated with worse prognosis [25]. However, none of these studies took the immune component of the microenvironment into consideration.

Importantly, the macrophage infiltration considered in the tumor databases is mainly derived from the tumor core, which is generally the most hypoxic area. It is possible that, depending on localization, macrophage might function in different ways. As our group and others reported, the macrophage inflammatory profile varies according to their location: adjacent tissue, invasive front or intratumor region [2,26]. It will therefore be interesting to assess in patients’ samples whether hypoxia influences other macrophage populations besides the ones from the core of the tumor, which is the main region represented in the TCGA patients’ samples.

Paradoxically, we found that in vitro hypoxia decreases the expression of macrophage antigen-presenting molecules, namely, HLA-ABC and HLA-DR, and of the co-stimulatory receptor CD86. These results are in line with other reports conducted with macrophage monocultures under hypoxia [27]. Nevertheless, antigen-presentation may still occur at the expense of other immune cells, such as dendritic cells.

This is the first study demonstrating that, although not affecting the percentage of phagocytic macrophages, colon cancer cells and hypoxia enhance macrophage phagocytic capacity. This characteristic might be important in vivo given the increase in necrosis, resulting in enhanced cell debris to collect. In addition, at the molecular level, this finding may suggest the inexistence of a functional anti-phagocytic SIRPα/CD47 system. In line with previous reports in other tumors, we found that hypoxic stimulation leads to an increase of CD47 levels on cancer cells [12,28]. In our system, the enhanced expression of this “don’t eat me” signal is counteracted by a decrease in macrophage SIRPα expression. In fact, it seems that less SIRPα expression is associated with increased phagocytosis. In agreement, we found that the percentage of SIRPα+ phagocytic macrophages significantly decreases at 1% O_2_, explaining the increased macrophage phagocytic capacity. Accordingly, other authors have described that silencing or blocking of SIRPα in RAW264.7 macrophages promoted the phagocytosis of osteosarcoma cancer cells [29]. Our findings regarding phagocytosis are divergent from previously reported work on breast cancer [12] but are in line with other results showing that macrophagic HIF1α enhances phagocytosis [30], suggesting that this might be a feature dependent on the cancer type. For the first time, we described that, in colon cancer, hypoxia impacts the SIRPα/CD47 axis through the decrease of macrophage SIRPα expression, which may favor their phagocytic capacity. Importantly, the molecular mechanisms associated with this apparent impairment of the SIRPα/CD47 axis need further elucidation.

Regarding the impact of macrophages and hypoxia on cancer cells, we found that despite the increased levels of glucose consumption and *SLC2A1* expression, there was no increase of lactate production, despite the increase in *LDHA* expression and acidosis. Previous reports have stated that lactate polarizes macrophages into an anti-inflammatory phenotype [31]. In our model, where no major alterations on lactate secretion were detected, we also did not find a clear-cut shift on macrophage phenotype. We found that hypoxia potentiated the already described ability of macrophage to induce cancer cell invasion [6]. The maintenance of this pro-tumor feature, despite the secretion of pro-inflammatory cytokines, had already been reported by our group, when macrophage and colon cancer cell co-cultures were treated with ionizing radiation [32].

Some authors have claimed that hypoxia leads to macrophage recruitment and polarization into an anti-inflammatory phenotype [33], while others have reported that hypoxia is not a major driver of macrophage polarization, but instead only triggers fine-tuning of metabolic genes in anti-inflammatory macrophages [34]. Others have described that *HIF1A* depleted macrophages exhibit increased anti-inflammatory markers [35]. In our model, we found alterations in both anti- and pro-inflammatory markers, and, at the secretome level, a shift into a pro-inflammatory phenotype is suggested. For the first time, we demonstrated that macrophages under the influence of colon cancer cells and hypoxia significantly decreased CCR7 expression, in line with other reports of macrophages monocultures [27]. Nevertheless, no impact in the pro-inflammatory markers IL-6 and TNF-α was observed. Interestingly, it has been recently described that HIF and IL-6 expression is increased in colorectal tumors, being both markers positively correlated [36]. In fact, we observed a slight increase in IL-6 secretion levels at 1% O_2_, although without statistical significance. In line with our findings, THP-1 macrophages under hypoxia exhibit decreased CD163 mRNA levels [37]; also being reported is that there is a decrease in CCL18 and IL-10 and an increase in IL-1β secretion in macrophages monocultures [27,38]. Accordingly, in our work, the analysis of CM of co-cultures combined with the total lysates of macrophages and cancer cells, evidenced that macrophages are the main producers of secreted IL-1β. While in macrophages, hypoxia is triggering increased IL-1β expression, on cancer cells, the opposite was observed. Overall, our results suggest that hypoxia triggers a decrease in macrophage anti-inflammatory pressure.

Importantly, we found that in *CD68*^High^ tumors, hypoxia is associated with the expression of pro-inflammatory cytokines, such as *IL1B*, *IL6,* and *TNF*, previously associated with the recruitment of other anti-tumor immune cells [18,19,20], suggesting the establishment of a pro-inflammatory TM. Interestingly, some authors described that hypoxia-associated anti-inflammatory macrophages repress T-cell activity [9], while others reported that (1) macrophages without HIF had less capacity to stimulate CD8 T-cell proliferation [39]; (2) hypoxia protected activated T-cells from death [40]; (3) CD8 T-cells with constitutive HIF expression delayed T-cell differentiation into effector cells but increased their cytotoxic function, through increased expression of granzyme B [41]. Notably, the *CD68*^High^*HIF1A*^High^ tumors are those presenting higher levels of infiltrated NK and cytotoxic T-cells, which could be related to the improved survival rate observed in these patients. In fact, these findings are in accordance with other reports demonstrating that colon tumors with higher T-cell infiltration are the ones associated with improved survival [42].

We hypothesize that the results described in this work might be particularly important in the CMS1 MSI-immune tumors, those characterized by an immune infiltrate mainly composed of Th1, NK, and cytotoxic T-cells, and with upregulation of proteins involved in immune response pathways [21,43]. Also, it was described that the number of tumors infiltrating macrophages is higher in MSI colon tumors, those associated with better prognosis [44]. Those tumors are generally associated with the proximal colon, and we confirmed that *HIF1A* was significantly upregulated in this region. Interestingly, it was only in those tumors located in the proximal colon that we found a positive correlation between *CD68* and *HIF1A*, and, furthermore, the survival curves associated with these two colon regions were clearly different from each other. It will be important to validate these results in bigger series of colon cancers, comparing distal and proximal tumors. Moreover, it is known that HIF1α represses DNA repair genes [45], which could account for an increased mutation burden and tumor immunogenicity. However, in our analysis, *HIF1A* expression could not be associated with mismatch repair protein expression. Interestingly, it is also known that mutant *BRAF*, a common event on CMS1 tumors, regulates HIF-1α expression in melanoma, affecting cell survival under hypoxic conditions [46]. Indeed, the colon cancer cases with *BRAF* mutation presented a significantly higher expression of *HIF1A*, while no differences were found on *KRAS* mutated cases. It will be interesting to further explore these associations among hypoxia, *BRAF* mutation and MSI status, and macrophage infiltration on colon tumors. In addition, given the documented relevance of the microbiome in cancer progression, the knowledge that there is a particular microbiota organization in proximal colon [47], and that colon bacterial products modulate HIF1-α expression and activation [48,49], it may also be important to explore the impact of the microbiome on macrophage–colon cancer cell crosstalk, within an hypoxic environment.

Despite the well documented influence of hypoxia in triggering different hallmarks of cancer, this work highlights that, depending on the component of the microenvironment, the tumor origin, and even on its localization, hypoxia can play a beneficial role in the combat against the tumor.

## 4. Materials and Methods 

### 4.1. Bioinformatic Analysis

#### TCGA

The Cancer Genome Atlas (TCGA; https://cancergenome.nih.gov/) dataset of colon carcinomas was analyzed, and for comparison purposes, gastric, breast ductal, and lung adenocarcinoma datasets were used. Survival plots were performed in R using survival and survminer packages. Median values were calculated in normal tissue and used as threshold to define “high” and “low” expression levels in cancer patients. The expression of genes was quantified as FPKM (fragments per kilobase million), which are provided by TCGA consortium. The number of individuals with information simultaneously on gene expression and survival were 136 for colon, 269 for gastric, 673 for breast ductal, and 52 for lung adenocarcinoma. Clinical and somatic mutation metadata were also provided by the TCGA consortium at https://gdc.cancer.gov/.

### 4.2. Oncomine

*CD68, HIF1A*, *CA9*, *LOX*, *IL1B*, *IL6*, *TNF*, *CD3D*, *NCAM*, *GZMB*, *CD8A,* and *TBX21* mRNA expression was assessed in the Bittner colon cancer dataset from Oncomine database (https://www.oncomine.org), encompassing a total of 374 cases. Categorization of patient samples was assigned into low (lowest 50%) and high (highest 50%) subgroups according to the log2 median-centered intensity levels of mRNA expression.

### 4.3. Ethics Statement

The buffy coats used for monocyte isolation are highly leukocyte-enriched waste-products that results from a whole blood donation of healthy blood donors. The isolation of immune cells from healthy blood donors was approved by the Centro Hospitalar Universitário São João Ethics Committee (protocol 90/19) after collecting each donor’s informed consent.

### 4.4. Human Monocyte Isolation and Macrophage Differentiation 

Human monocytes were isolated from buffy coats from healthy blood donors, as previously described (6). Isolated cells were resuspended in RPMI1640 supplemented with 10% fetal bovine serum (FBS, Biowest, Nuaillé, France) and 100U/mL penicillin and 100 μg/mL streptomycin (PS, Invitrogen, Carlsbad, CA, USA). Then, 1.2 × 10^6^ monocytes were plated per 6-well culture plates and differentiated into macrophages for 10 days. In the first 7 days, macrophages were supplemented with M-CSF (50 ng/mL, ImmunoTools, Friesoythe, Germany). Cells were maintained at 37 °C and 5% CO_2_ humidified atmosphere.

### 4.5. Cancer Cell Culture

RKO colon cancer cell line, from American Type Culture Collection (ATCC), was cultured in RPMI1640 (Invitrogen), supplemented with 10% FBS and 1% PS, and maintained at 37 °C and 5% CO_2_ humidified atmosphere. 

### 4.6. Macrophage and Cancer Cell Indirect Co-Cultures

For co-cultures, 1 × 10^5^ RKO cells were seeded into 6-well-plate permeable transwell inserts (PET inserts with 1-μm pore, Corning, Corning, NY, USA) positioned on top of a macrophage culture, 10 days after monocyte isolation. Co-cultures between macrophages and cancer cells were maintained in complete RPMI medium for 72 h at 37 °C, at 20% O_2_ or 1% O_2_ (94% N_2_), 5% CO_2_ humidified atmosphere.

### 4.7. RNA Extraction, cDNA Preparation, and Quantitative Real-Time PCR Analysis

Total RNA was extracted using TRIzol reagent (Invitrogen), according to the manufacturer’s instructions. cDNA was synthesized using 1 μg of RNA, using NZY M-MuLV Reverse Transcriptase enzyme (NZYTech, Lisboa, Portugal), according to the manufacturer’s instructions. Quantitative real-time PCR (qRT–PCR) was performed with Kapa Probe Fast qPCR Master Mix (Kapa Biosystems, Roche, Basel, Switzerland) using the probes to *ACTB* (Hs01060665), *CA9* (Hs00154208), *LDHA* (Hs.PT.58.22929122), and *SLC2A1* (Hs.PT.58.25872862) (Applied Biosystems, Foster City, CA, USA). Relative mRNA expression of the target genes was normalized to the levels of the housekeeping gene, using the comparative ΔΔCt method. ACTB was used as a housekeeping gene to RKO cells.

### 4.8. Viability Assay

Macrophage and cancer cell viability was measured by resazurin reduction assay. After 3 days of co-culture, resazurin redox dye (0.01 mg/mL, Sigma-Aldrich, St.Louis, MO, USA) was added (1/10 of the total volume of culture medium) to cell culture, incubated for 4 h at 37 °C and 5% CO_2_, and the fluorescence intensity was measured at 530/590 nm in a Synergy Mx fluorometer.

### 4.9. Flow Cytometry

Macrophages and cancer cells were incubated with accutase (eBioscience, Affymetrix, Santa Clara, CA, USA) at 37 °C for 30 min and harvested by gently scraping. Cells were washed and resuspended in FACS buffer (PBS, 2% FBS (Biowest), 0.01% sodium azide) containing appropriate conjugated antibodies, and stained in the dark for 40 min at 4 °C. Macrophages were immunostained with the following antibodies: anti-human CD14-APC (clone MEM-12), CD14-PE/FITC (clone 18D11), CD14-PerCP/Cy5.5 (clone OFC14D), CD86-FITC (clone BU63), HLA-ABC-PE (clone W6/32), HLA-DR-PE/FITC (clone MEM-12) (Immunotools), CCR7-PerCP/Cy5.5 (G043H7), SIRPα-APC (clone SE5A5) (Biolegend, San Diego, CA, USA), CD163-PE (clone GHI/61) (BD Bioscience, Franklin Lakes, NJ, USA). Cancer cells were stained with anti-human CD47-FITC (clone MEM-122) (Immunotools). Unstained cells and single stained with antibodies or with the respective isotype IgGs were used as control. After additional washing steps, cell fluorescence was acquired on a FACS Canto Flow Cytometer (BD Biosciences) using FACS Diva Software. Data analysis was performed with FlowJo software.

### 4.10. Phagocytosis Assay 

Colon cancer cells were grown for 72 h at 1% O_2_, and after this period, suspension of 1 × 10^7^ cells/mL was stained with 2.5 μM CellTrace™ CFSE (Invitrogen), according to the manufacturer’s protocol. Macrophages that were previously indirectly co-cultured with cancer cells, for 72 h, were incubated in serum-free medium for 2 h before adding 2.4 × 10^6^ CFSE-labeled cancer cells (1 macrophage:2 cancer cells). After coculture for 2 h at 37 °C, macrophages were harvested and evaluated by flow cytometry, with anti-CD14 and anti-SIRPα antibodies. Phagocytosis was calculated as the percentage of CD14+CFSE+ cells among total CD14+.

### 4.11. Enzyme-Linked Immunosorbent Assay (ELISA) 

Cytokine levels were measured in the CM of mono and co-cultures by ELISA to TGF-β, TNF-α, IL-10, IL-1β, IL-6 (Biolegend), and CCL18 (Abcam, Cambridge, UK) according to the manufacturer’s instructions. Concentrations were normalized to the CM total protein concentration, which was measured using the DCProtein assay kit (BioRad, Hercules, CA, USA), following the manufacturer’s instructions.

### 4.12. Proliferation Assay

Cell proliferation was determined through the use of the Click-iTTM Plus EdU Flow Cytometry Assay Kit (Invitrogen), according to the manufacturer’s instructions. RKO cells cultured in 6-well plates without EdU treatment were used as a negative control, while RKO treated with EdU was used as a positive control.

### 4.13. Cell Death Quantification Assay

Cell death was measured using the FITC Annexin V Apoptosis Detection kit (BD Pharmigen, BD Bioscience, Franklin Lakes, NJ, USA) according to the manufacturer’s instructions. Untreated RKO cells cultured in a 6-well plate were used as a negative control, and cells treated with H_2_O_2_ were used as a positive control.

### 4.14. Glucose Quantification Assay

Glucose levels were measured in the CM of the co-cultures using the enzymatic colorimetric kit, Glucose Assay Kit (Roche), following the manufacturer’s instructions. The standard curve for glucose quantification was done using serial dilutions of RPMI, with known glucose concentration.

### 4.15. Lactate Quantification Assay

An enzymatic colorimetric kit (Spinreact, Girona, Spain) was used to determine lactate concentration in co-cultures CM, according to the manufacturer’s instructions. 

### 4.16. pH Measurement

The CM pH was measured using the Electro Kinetic Analyzer (EKA) (Anton Paar, Graz, Austria).

### 4.17. Matrigel Invasion Assay

Invasion was measured using Matrigel-coated invasion inserts of 8μm pore (Corning), according to the manufacturer’s instructions. In the upper compartment, 2.5 × 10^4^ RKO cells were cultured at 20% O_2_ or 1% O_2_ conditions, either alone or stimulated with macrophages cultured at 20% O_2_ or 1% O_2_ at the lower compartment. Cells were allowed to invade for 24 h at 37 °C, at 20% O_2_ or 1% O_2_, respectively. After this period, the inserts were washed with PBS and fixed in 4% PFA for 20 min at RT, and the non-invading cells in the upper compartment of the inserts were removed. The filters were mounted in Vectashield Mounting Medium with DAPI (Vector Laboratories, Burlingame, CA, USA), and the invading cells were visualized and counted with a Leica DM2000 fluorescence microscope (Leica Microsystems, Wetzlar, Germany).

### 4.18. Western Blot

Cell lysates were prepared with Rippa buffer (50 mM Tris HCl pH = 7.5; 1% NonidetP (NP)-40; 150 mM NaCl and 2 mM EDTA) with proteases/phosphatases inhibitors cocktails (Sigma-Aldrich). Protein concentration was determined using the DCProtein assay kit (BioRad) and 20 μg of protein were loaded and run in a 12.5% SDS-polyacrylamide gel. Afterwards, gels were transferred to nitrocellulose membranes (GE Healthcare, Chicago, IL, USA), blocked with 5% non-fat powder milk in PBS + 0.1% Tween-20 (PBS-T 0.1%) for 30 min, and incubated overnight with primary antibodies rabbit anti-IL-1β (GeneTex, Irvine, CA, USA), and mouse anti-Hsc-70 (Santa Cruz, Dallas, TX, USA). HRP-conjugated anti-mouse and anti-rabbit (GE Healthcare, Chicago, IL, USA) were used as secondaries antibodies. Membranes were incubated with Clarity Western ECL Substrate (BioRad) for signal detection. Bands intensity measurements were performed with ImageJ.

### 4.19. Statistical Analysis 

Data were expressed as the mean with standard deviation (SD), and collected from at least three independent experiments with macrophages from different donors. The normality of the distribution was evaluated using the Kolmogorov–Smirnov test. For comparison between two independent groups, *t*-test (paired, Wilcoxon, and Mann–Whitney) was used. For comparison among four independent groups, one-way ANOVA (RM one-way ANOVA, Friedman, and Kruskal–Wallis), with Dunn’s multiple comparison was used. Correlations were tested with the Spearman test and considered moderate positive when Spearman Rs were between 0.2500 and 0.3500, and strong positive when Spearman R > 0.3500. Survival curves were analyzed with the log-rank (Mantel–Cox) test. Statistical tests were performed in GraphPad Prism 8. Differences were considered significant at *p* < 0.05.

## 5. Conclusions

Our results demonstrate the importance of considering hypoxia as a major player on the tumor microenvironment modulation. In particular, we found that in colon tumors with high macrophage infiltration, hypoxia is associated with better prognosis, possibly due to the pro-inflammatory pressure induced by macrophages, which could potentiate the recruitment of other anti-cancer immune cells. This phenomenon appears to be tissue-specific and even dependent on tumor localization within the colon. 

Altogether, these data underlie the need to consider the specificities of each tumor microenvironment, since the particular context in which cancer cells are enclosed can have a major impact in disease prognosis and the selection of therapeutic options.

## Supplementary Materials

The following supplementary figures are available online at https://www.mdpi.com/2072-6694/12/4/818/s1, Figure S1: Experimental setup and confirmation of cellular response to hypoxia; Figure S2: Gating strategy for flow cytometry analysis; Figure S3: The *CD68^High^HIF1A^High^* population is associated with increased recruitment of cytotoxic T-cells; Figure S4: Western blot.
