# Supplementary material for: Hypoxia and Macrophages Act in Concert Towards a Beneficial Outcome in Colon Cancer
Source: Cancers (Basel). 2020 Mar 28;12(4):818. doi: 10.3390/cancers12040818 (PMC7226541; doi:10.3390/cancers12040818)
Supplement: Supplementary file 1 [file cancers-12-00818-s001.pdf]

Supplementary Materials:

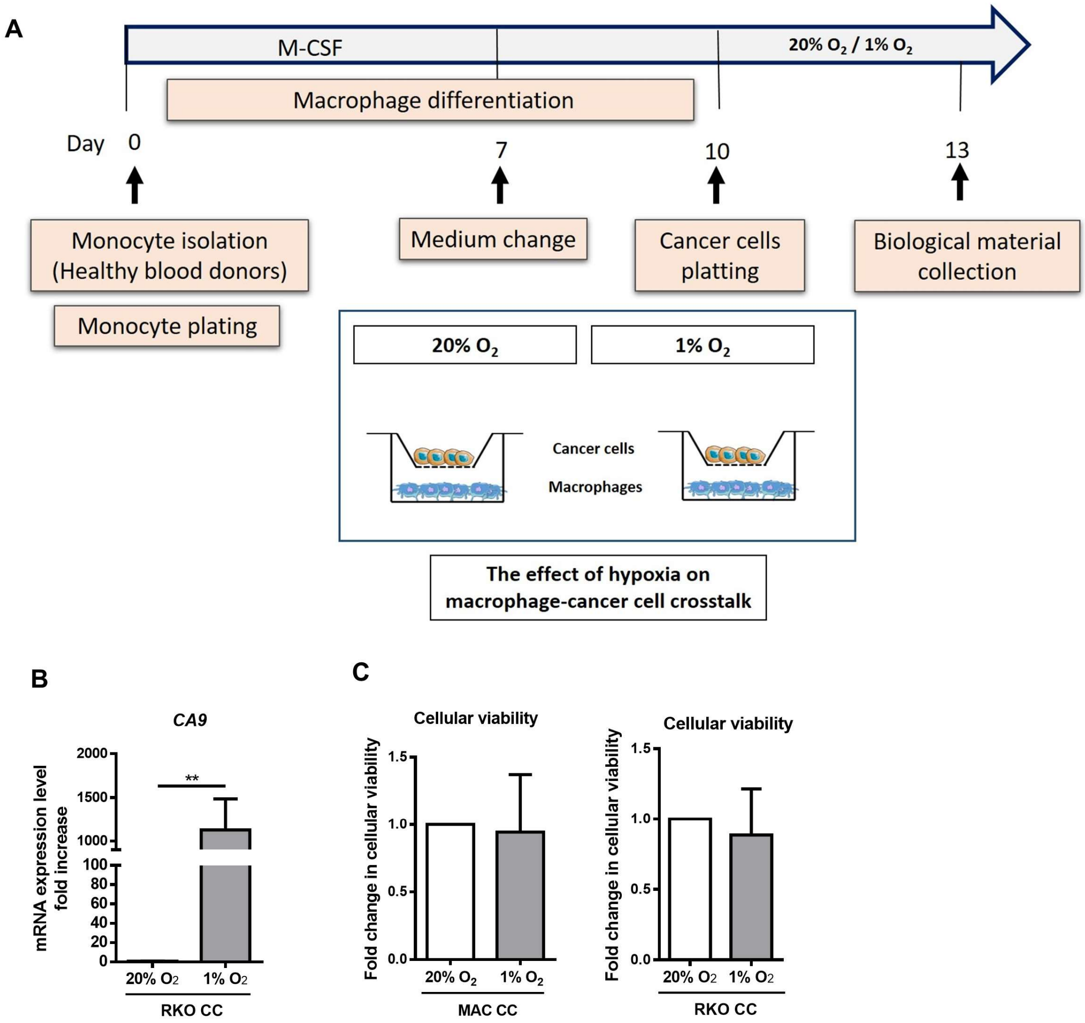

Figure S1. Experimental setup and confirmation of cellular response to hypoxia. (**A**) Schematic representation of the experimental setup. (**B**,**C**) Macrophages were indirectly co-cultured with RKO, at 20% or 1% O_2 for 72 h, and (**B**) cancer cells *CA9* mRNA levels, a hypoxic marker, were measured by qRT-PCR. Relative expression changes are presented as fold variation CA9/*ACTB* relatively to 20% O_2 condition. (**C**) Co-cultured macrophages (MAC CC) and cancer cells (RKO CC) viability was measured by resazurin assay. Relative changes are presented as fold variation relatively to 20% O_2 condition. Graphs represent the mean values with standard deviations of 8 independent experiments. The statistical tests Wilcox or paired t-test were used. ** $p < 0.01$.

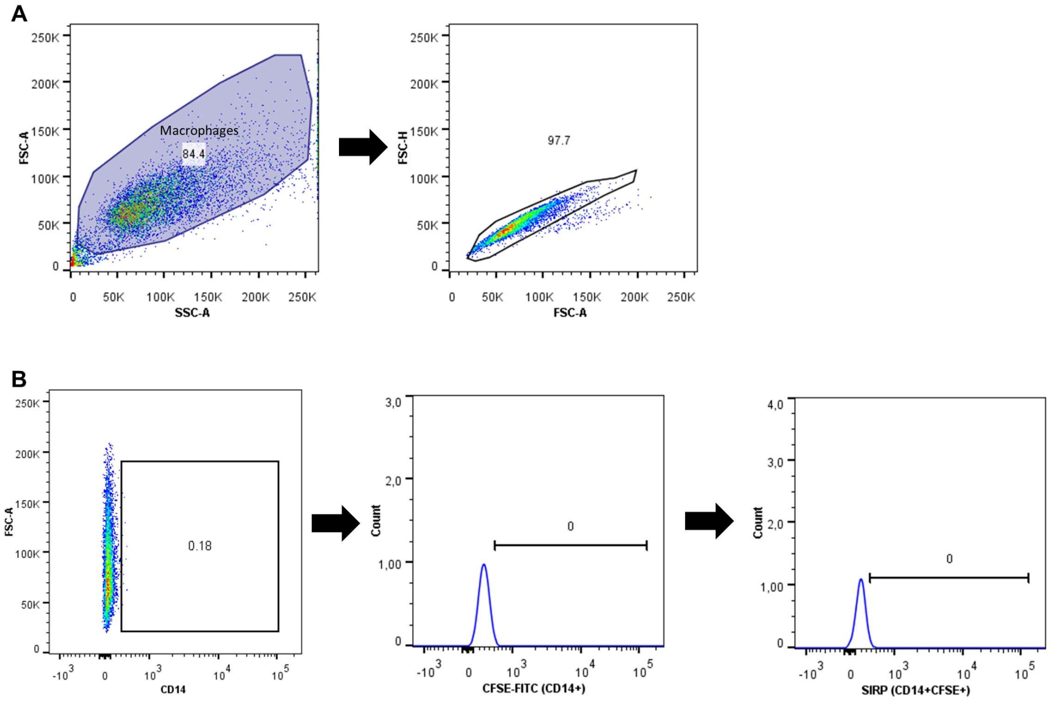

Figure S2. Gating strategy for flow cytometry analysis. (**A**) The scatter plots exhibit a representative image of the gating strategy created with FlowJo software for flow cytometry analysis. FSC-A/SSC-A exemplifies the distribution of cells in the light scatter based on cell size and granularity, respectively; FSC-A/FSC-H represents the single cells of the previously selected population. (**B**) For the phagocytosis experiments, macrophages were identified using $CD14^+$, and within this population the $CFSE^+$ population was determined. Within the $CD14^+CFSE^+$ population, $SIRP\alpha^+$ cells were quantified.

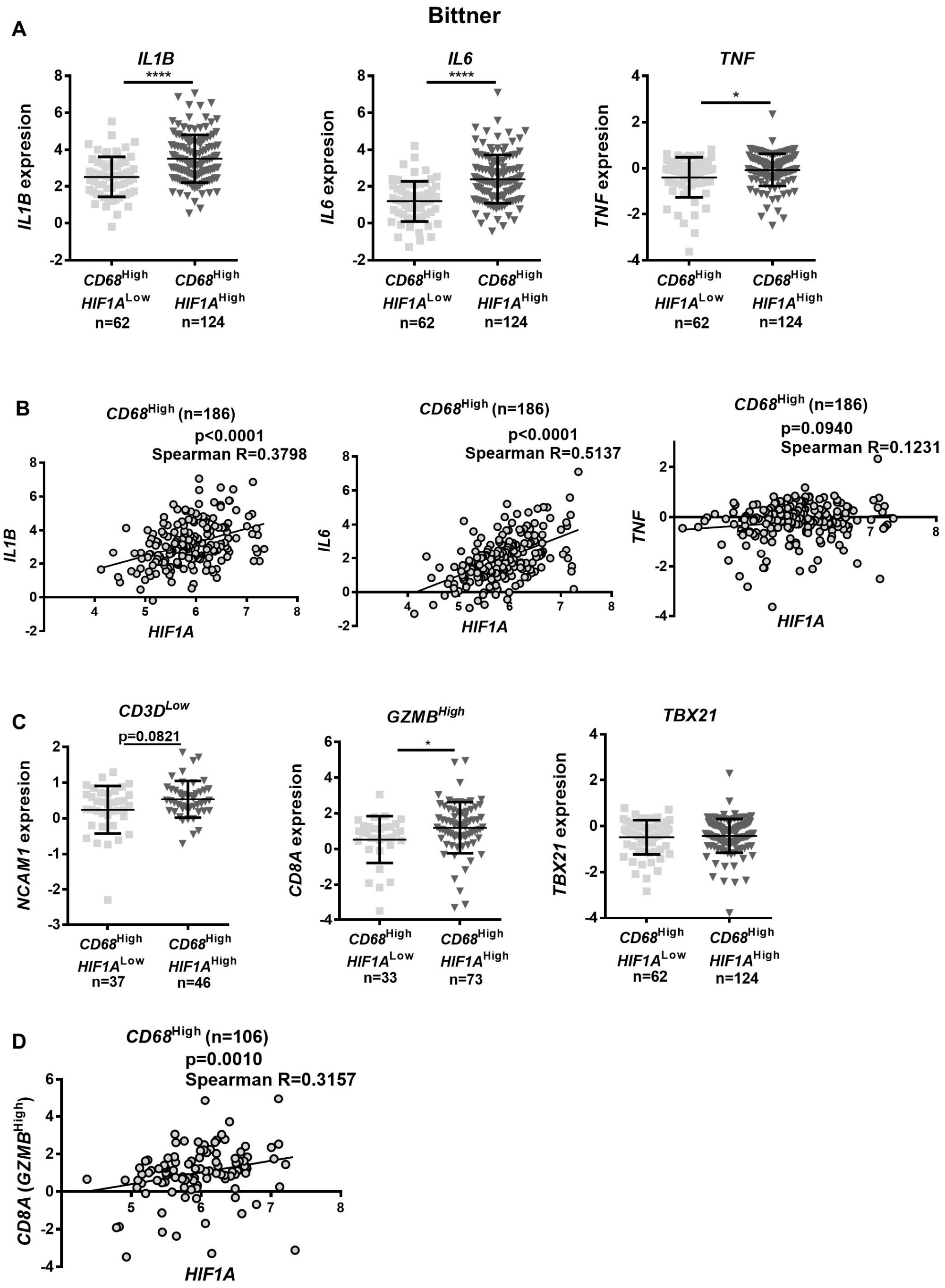

Figure S3. The *CD68*^{High}*HIF1A*^{High} population is associated with increased recruitment of cytotoxic T cells. Microarray expression data were downloaded from the Bittner colon cancer cohort from the Oncomine database. (**A**) Expression of *IL1B*, *IL6*, and *TNF* was evaluated within the *CD68*^{High} population, between *HIF1A*^{Low} and *HIF1A*^{High}, according to the median levels of *CD68* and *HIF1A* expression in the tumors. (**B**) The correlations between *IL1B*, *IL6*, *TNF* and *HIF1A* expression were assessed within the *CD68*^{High} population. (**C**) *NCAM1*, *CD8A*, and *TBX21* expression was evaluated within the *CD68*^{High} population, between *HIF1A*^{Low} and *HIF1A*^{High}, according to the median levels of *CD68* and *HIF1A* expression in the tumors. *NCAM1* levels were evaluated within the *CD3D*^{Low} population, *CD8A* levels were evaluated within the *GZMB*^{High} population. *CD3D*^{Low} and *GZMB*^{High} populations were established according to the median levels of *CD3D* and *GZMB* expression in the tumors. (**D**) The correlation between *CD8A(GZMB*^{High}*)* and *HIF1A* was assessed within the *CD68*^{High} population. Mann-Whitney or unpaired t-test statistical tests were used to compare expression

between groups. Spearman statistical test was used to assess correlation, which was considered moderate positive when Spearman R were between 0.2500 and 0.3500, and strong positive when Spearman R > 0.3500. **** $p < 0.0001$; *** $p < 0.001$; * $p < 0.05$. The p values between 0.05 and 0.1 were presented, and considered a tendency.

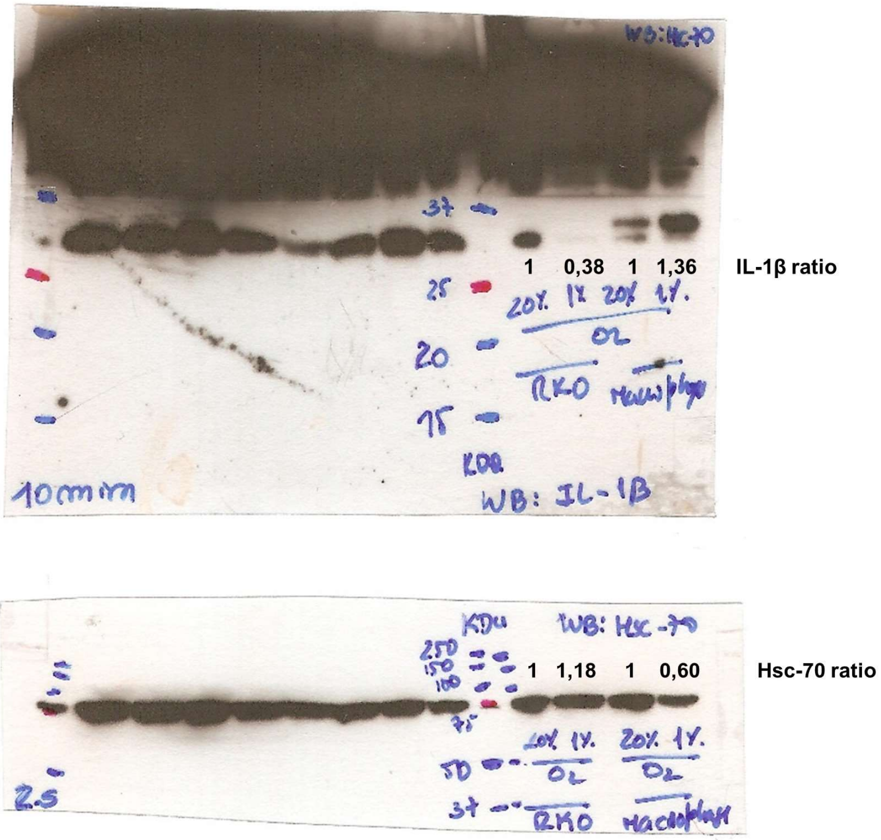

Western Blot

Figure S4. Western Blot raw data.
